# Cutaneous neurofibromas in the genomics era: current understanding and open questions

**DOI:** 10.1038/s41416-018-0073-2

**Published:** 2018-04-26

**Authors:** Robert J. Allaway, Sara J. C. Gosline, Salvatore La Rosa, Pamela Knight, Annette Bakker, Justin Guinney, Lu Q. Le

**Affiliations:** 1 Sage Bionetworks, Seattle, WA 98109 USA; 2 Children’s Tumor Foundation, New York, NY 10005 USA; 3 Department of Dermatology, Simmons Comprehensive Cancer Center and the Neurofibromatosis Clinic, UT Southwestern Medical Center, Dallas, TX 75390 USA

**Keywords:** Skin manifestations, Medical genomics

## Abstract

Cutaneous neurofibromas (cNF) are a nearly ubiquitous symptom of neurofibromatosis type 1 (NF1), a disorder with a broad phenotypic spectrum caused by germline mutation of the neurofibromatosis type 1 tumour suppressor gene (*NF1*). Symptoms of NF1 can include learning disabilities, bone abnormalities and predisposition to tumours such as cNFs, plexiform neurofibromas, malignant peripheral nerve sheath tumours and optic nerve tumours. There are no therapies currently approved for cNFs aside from elective surgery, and the molecular aetiology of cNF remains relatively uncharacterised. Furthermore, whereas the biallelic inactivation of *NF1* in neoplastic Schwann cells is critical for cNF formation, it is still unclear which additional genetic, transcriptional, epigenetic, microenvironmental or endocrine changes are important. Significant inroads have been made into cNF understanding, including *NF1* genotype–phenotype correlations in NF1 microdeletion patients, the identification of recurring somatic mutations, studies of cNF-invading mast cells and macrophages, and clinical trials of putative therapeutic targets such as mTOR, MEK and c-KIT. Despite these advances, several gaps remain in our knowledge of the associated pathogenesis, which is further hampered by a lack of translationally relevant animal models. Some of these questions may be addressed in part by the adoption of genomic analysis techniques. Understanding the aetiology of cNF at the genomic level may assist in the development of new therapies for cNF, and may also contribute to a greater understanding of NF1/RAS signalling in cancers beyond those associated with NF1. Here, we summarise the present understanding of cNF biology, including the pathogenesis, mutational landscape, contribution of the tumour microenvironment and endocrine signalling, and the historical and current state of clinical trials for cNF. We also highlight open access data resources and potential avenues for future research that leverage recently developed genomics-based methods in cancer research.

## Neurofibromatosis type 1: aetiology and symptoms

Neurofibromatosis type 1 (NF1) is a genetic disorder that affects 1:2600–1:4500 live births.^1,2^ The disease has nearly complete penetrance, and patients present with a diverse spectrum of manifestations. Hallmark traits of NF1 include café-au-lait spots, cutaneous neurofibromas (cNFs, or dermal neurofibromas), plexiform neurofibromas (pNFs) and malignant peripheral nerve sheath tumours (MPNSTs), among other symptoms.^3,4^ Although the disorder was first clinically described in the 1800s, it was not until much later that the neurofibromatosis type 1 gene (*NF1*) was identified.^5–8^ NF1 is caused by inherited or de novo germline mutations in the *NF1* tumour suppressor gene, and it is thought that somatic loss-of-function of the second allele results in the development of tumours such as pNFs, MPNSTs and cNFs.

**Fig. 1:**
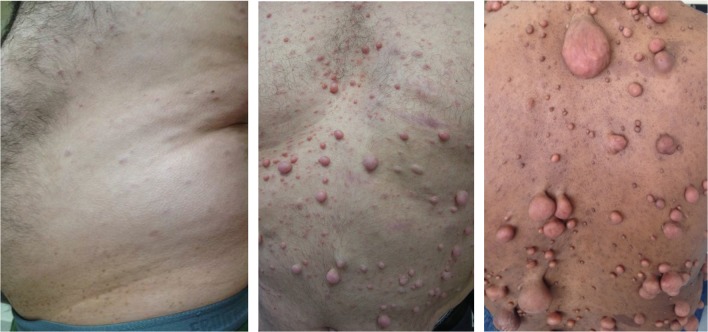
Cutaneous neurofibromas in NF1 patients. Cutaneous neurofibromas occur in nearly all NF1 patients, but they present with great diversity in both tumour frequency and tumour size.^13–16^ These tumours represent one of the most challenging burdens for neurofibromatosis type 1 patients.^15,16,19^ Images are used with patient permission

NF1-linked tumours present with differing frequencies across NF1 patients. PNFs are benign nerve sheath tumours that occur in ~40% of NF1 patients.^9,10^ Notably, pNFs have the capacity to develop into MPNSTs, which affect 6–13% of NF1 patients.^11,12^ Unlike pNFs, cNFs do not progress to malignancy, but they are observed in >99% of adult patients and can range widely in both size and number (Fig. 1).^13–16^ While these tumours are generally observed during puberty and pregnancy, cNFs are sometimes observed before 5 years of age.^17,18^ These benign cNF tumours can cause itching, pain and a cosmetic burden that has been linked with psychosocial challenges.^15,19^ Therefore, NF1 patients often identify the cNF tumours as their greatest burden. Additionally, treatment options for cNF are limited to elective surgical approaches, which presents a challenge for patients with thousands of tumours. The term 'cutaneous neurofibroma' (cNF) is used in this review to describe benign neurofibromas that are found exclusively within the cutaneous dermis layer; cNFs are therefore also often called dermal neurofibromas. In addition, 'benign neurofibroma' can also refer to non-cNFs including benign subcutaneous, internal, diffuse or plexiform neurofibromas. Currently, there are no well-defined subtypes of cNF that represent stages of tumour growth or phenotypically distinct cutaneous neurofibromas; elucidating these subtypes is an important challenge for the field.

While many studies have contributed to our understanding of cNF, there are still large gaps that remain to be explored. In this review, we summarise the present understanding of cNF pathogenesis including recent studies that propose a putative tumour cell of origin, the identification of mutations in NF1 and other genes in cNF, the role of the tumour microenvironment and endocrine signalling in cNF, and past, recent and ongoing clinical trials for cNF. We also propose the application of genomics-oriented approaches for future investigations of cNF.

## Histology, origin and pathogenesis of cNF

cNFs manifest as small (2 mm–3 cm), circumscribed tumours that associate with nerves in the skin.^14,20^ Clinically, they can undergo a rapid initial proliferative phase but then quickly become quiescent with extremely slow to no growth.^21^ Their diverse composition is similar to that of the nerve sheath, consisting of Schwann cells, perineural cells, fibroblasts and a collagenous matrix.^14,22,23^ cNFs also contain an infiltrating immune cell population that is comprised of macrophages and mast cells.^23–25^ Studies in mouse models have identified a critical role for mast cells in pNF formation, but it is unknown whether mast cells and macrophages contribute to the growth of cNF tumours.^23,24,26^ Histologically, cNFs share similar markers to other NF1-associated nerve tumours such as pNFs, and can be identified by wavy nuclei, S100 positivity and the expression of collagen IV, Sox10, CD34 and CD44. Of note, S100 and Sox10 can be used as diagnostic markers.^20,27,28^

The cNF-tumour-initiating event in humans is currently unknown and the cell of origin has yet to be identified; however, studies in mice have led to the identification of Schwann-cell-like skin-derived precursor (SKP) cells as the likely murine cNF tumour cell of origin. Wu and colleagues observed cNF-like tumours in the *Nf1*^*fl/fl*^; *DhhCre* mouse model, and proposed that these tumours may originate from neural-crest-derived cells in the hair follicles.^29^ Shortly thereafter, Le et al. found that cNFs can originate from a population of neural-crest-derived progenitors residing in the dermis, identified as SKPs.^30^ These authors generated CMV-CreER^T2^;Nf1^flox/-^;ROSA26 mice and ablated *Nf1* in the presumed cNF cell of origin in the skin; this was achieved with cutaneous application of tamoxifen.^30^ They observed the formation of cNFs at the tamoxifen application sites 6–7 months later, suggesting that the cNF cell of origin resides in the skin. Nf1-deficient SKPs also have the capacity to form pNFs or cNFs, which appears to be contingent on their local microenvironment, and these cells exhibit the same properties as the embryonic Schwann cell progenitors that give rise to pNFs.^29,30^ These observations revealed that loss of *Nf1* gene expression in SKPs is required, but not sufficient, for neurofibroma development, and revealed critical roles for the tumour microenvironment. Consistently, when *Nf1*^*−/*−^ SKPs were autologously implanted intradermally, they efficiently gave rise to cNFs in mice that were pregnant at the time of implantation.^30^ This suggested that the hormonal milieu can facilitate induction of cNF development from *Nf1*-deficient SKPs in the skin. More recent work demonstrated that implantation of *Nf1*^*−/−*^ SKPs in sciatic nerve tissue, but not subcutaneous implantation in unprimed athymic mice, resulted in neurofibroma formation, further indicating that the microenvironment may be a key regulator of cNF development.^24^

It is not yet known whether human cNFs also originate from SKPs or other cell populations. Longitudinal natural history studies are currently underway that may address this gap.^31,32^ Considering this information, the natural history of cNF is poorly understood for multiple reasons: patients can develop many cNFs throughout life, cNF burden varies greatly among NF1 patients and the precise sequence and nature of all cNF-initiating events is unknown. This limited understanding of cNF natural history is one obstacle in generating models of the disease but there are several other challenges to developing translationally relevant models of cNF. cNFs are slow-growing benign tumours that may take more than a year to develop, possibly beyond the useful lifespan of commonly used experimental organisms. In addition, although the Nf1^−/−^ Schwann cell is the neoplastic cell in cNFs, human cNFs involve multiple other cell types and a complex extracellular matrix, making it challenging to recapitulate the human disease in vivo as well as in vitro. Finally, in vivo and in vitro cNF models must originate from the same cell of origin as the human tumour, they must be driven to tumourigenesis by the same genetic drivers (intrinsic factors), undergo similar downstream transcription profiles, develop within a similar tumour microenvironment (extrinsic factors) and must be robust for preclinical screening. Many of these key elements of cNF remain to be clearly defined.

### Role of NF1 mutations in the phenotype of cNF

**Fig. 2:**
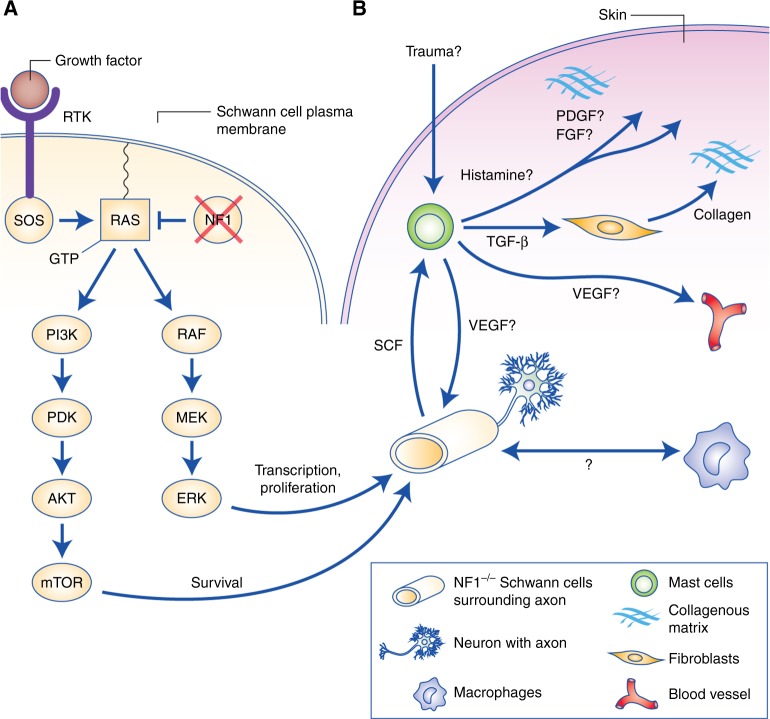
The RAS signalling cascade and tumour microenvironment in cNF. **a** Loss of *NF1* reduces the ability of Ras to hydrolyse GTP and shift from an active to a GDP-bound inactive state. Consequently, Schwann cells lacking NF1 have increased proliferation and altered transcription (RAF-MEK-ERK signalling) and increased prosurvival signalling (PI3K-mTOR). **b** The cNF microenvironment is composed of NF1-deficient Schwann cells, mast cells, fibroblasts, macrophages and neurons, among other cell types. While roles for some of these cell types have been studied or hypothesised, particularly with regard to mast cells, the bulk of these cellular interactions are poorly understood or unstudied

The *NF1* gene product, neurofibromin, plays an important role in regulating cellular transcription, proliferation and survival (Fig. 2a). Ligand-activated receptor tyrosine kinases drive Ras activation by signalling via Ras guanidine exchange factors, such as SOS;^33^ however, NF1 is a Ras GTPase activating protein, and thus facilitates Ras inactivation.^33,34^ In cells lacking NF1, Ras activation is not inhibited by NF1, which in turn induces transcriptional changes and proliferative signalling via the RAF-MAP kinase pathway, and prosurvival signalling via the PI3K-mTOR axis.^33,35^ Consequently, loss or mutation of *NF1* is an important step in NF1 patient tumourigenesis. Of note, next-generation sequencing has recently demonstrated that the *NF1* gene is frequently mutated in sporadic cancers not usually observed in NF1 patients,^36^ such as conjunctival melanoma.^37^

The mutational landscape of the *NF1* gene, however, is more diverse in NF1 patients; germline mutations are observed across the entire 3.5 kb *NF1* sequence.^6–8,38,39^ In contrast to oncogenic mutations, which often occur at specific loci and result in hyperactivation of the gene product, inactivating mutations observed in tumour suppressor genes such as *NF1* are not site specific. For example, a survey of germline *NF1* mutations in 189 NF1 patients determined that 45% of patients harboured one of 38 recurrent *NF1* mutations detected in this study (for example, *NF1* c.910C>T, *NF1* c. 1885G>A and *NF1* c.6792C>A), while 55% of patients had a unique *NF1* mutation.^38^ Other studies have similarly identified many novel germline *NF1* mutations across the length of the *NF1* coding region with no evidence of mutational hotspots.^40,41^ One database, the Leiden Open Variation Database – *NF1* (https://databases.lovd.nl/shared/genes/NF1), has identified over two thousand unique germline *NF1* variants.^39^

In addition to point mutations, germline *NF1* loss can be caused by microdeletion or pathogenic intronic mutations.^42,43^ The relationship between germline *NF1* microdeletion and cNF is somewhat clearer, compared with our understanding of the role of germline and somatic intragenic *NF1* mutations. Studies suggest that germline *NF1* microdeletions guide cNF development; cNFs that form in *NF1* microdeletion patients do not exhibit somatic loss of heterozygosity (LOH) of the second *NF1* allele, but instead typically contain point mutations in *NF1*.^44^ *NF1* microdeletion patients generally exhibit more severe symptoms than the general NF1 patient population, an effect attributed to the deletion of one or many modifying genes neighbouring *NF1*, such as *CRLF3, ATAD5, OMG, RAB11FIP4, SUZ12, LRRC37B* and several others.^45,46^ Generally, these *NF1* microdeletions fall into one of four categories: type 1 (1.4 Mb, 70–80% of deletions), type 2 (1.2 Mb, >10% of deletions), type 3 (1.0 Mb, 1–4% of deletions) and atypical (non-standard, 8–10% of deletions).^46^ Type 1 and type 3 deletions are generally germline mutations, type 2 deletions are generally somatic and atypical microdeletions may be germline or somatic.^46^ Furthermore, the type of microdeletion present appears to impact cNF burden. For example, 50% of *NF1* microdeletion syndrome patients with a type 1 deletion exhibit a high cNF burden (>1000 cNFs),^43,47^ while at least one atypical microdeletion (a 2.7 Mb region spanning from intron 21 of *NF1* to intron 1 of *ACCN1*) is associated with a complete absence of cNFs, despite the fact that this microdeletion partially overlaps with the typical (type 1, 2 and 3) microdeletion regions.^45,46^ However, other patients with similar atypical deletions still present with cNF.^45^ Collectively, these observations suggest that critical genes or epigenetic regulatory elements exist in the *NF1* microdeletion region, and that unresolved complexity remains, particularly regarding the impact of *NF1* microdeletions on cNF presence.

*NF1* function can also be impaired by post-transcriptional alterations such as alternative splicing, microRNA-mediated repression of the transcript or excessive proteasomal degradation of the protein.^48–50^ Consequently, detection of *NF1* loss-of-function events and clarification of the *NF1* genotype–phenotype relationship are existing challenges to the field.^51^ Some studies have started to characterise the genotype–phenotype relationship between *NF1* and cNFs, and these further suggest that there are a wide range of somatic *NF1* mutations associated with cNF.^52–54^ For example, Upadhyaya and colleagues identified 77 distinct somatic *NF1* mutations (53 of which were not previously described) in a study of 109 cNFs from 46 NF1 patients, of which 25/109 (~23%) cNFs exhibited *NF1* LOH.^54^ Another study by the same group identified *NF1* LOH in 22/89 (25%) of cNFs tested, and somatic *NF1* mutations in 57/89 (64%) of cNFs from three patients.^53^ The impact of most *NF1* somatic intragenic mutations on cNF characteristics is currently unclear. Similarly, it is unknown what effect intragenic germline *NF1* mutations have on cNF, with the exception of two mutations (*NF1* c.2970-2972delAAT and *NF1* c.5425C>T), which are associated with a lack of cNFs.^55,56^ *NF1* c.5425C>T may disrupt the structure of the pleckstrin homology-like domain of NF1, whereas *NF1* c.2970-2972delAAT is hypothesised to be a hypomorphic mutation.^55,57^ It is not currently understood why these mutations do not induce cNF formation.

Although it is not yet known for most clinical cases how *NF1* genotype correlates with cNF phenotype, one possibility is a Ras-signalling-dependent growth mechanism. Mice with tissue-specific expression of oncogenic Ras (N-Ras^G12V^) developed cNFs around 3 months post-birth, among other cutaneous symptoms of neurofibromatosis, suggesting that these phenotypes are caused by Ras activation.^58^ However, to the best of our knowledge, Ras mutations are not observed in either NF1-linked or sporadic cNFs. Considering this, experiments that examine the functional consequence of specific *NF1* variants on Ras activity may help clarify the *NF1* genotype–phenotype relationship.^54,59^ Perplexingly, germline inactivation of *Spred1*, which also results in Ras hyperactivation and the NF1-like disorder Legius syndrome, is not associated with the formation of cNFs.^60^ This indicates that there are unresolved mechanistic differences between *NF1* and *SPRED1* loss-of-function, and perhaps other modifiers that are requisite for cNF formation. Recent efforts using machine learning approaches, targeted sequencing panels for genes involved in RASopathies (diseases that are a consequence of Ras pathway dysregulation, such as NF1), and open access data resources are attempting to address these challenges.^61–63^ Specifically, these approaches are leveraging recent technological advances to assess *NF1* loss-of-function and the relationship between germline or somatic *NF1* mutations with the resulting phenotype. Way and colleagues trained a machine learning method to predict NF1 protein content, Bhoj et al. utilised a RASopathy sequencing panel in tandem with clinical examination to assess the presence of RASopathies, and Gosline et al. assembled the first public database of exome sequencing, RNA-seq and SNP array data for cNF.^61–63^ By integrating approaches such as these into cNF research, the field may be able to build upon prior studies to identify more accurate genotype–phenotype associations.

### Genetic perturbation beyond the *NF1* gene: impact on cNF development

Beyond the *NF1* gene, cNFs are thought to have a low somatic mutation burden when compared with most cancers. Research comparing exome sequencing data from seven distinct cNF tumours to blood and skin samples obtained from a single patient did not identify somatic mutations in 5/7 cNFs, beyond a second-hit somatic *NF1* mutation.^52^ The other two contained variants in *HMCN1* and *CEP131* (*AZI1*), respectively.^52^ A more recent study of exome data from three growing and three stable cNFs supports the hypothesis that these tumours are genomically quiet.^64^ Each tumour contained 1–11 somatic mutations, but no correlation was found between the type of variants and growth. Another study identified several recurrent somatic mutations in NF1-linked and sporadic cNFs, including *MAML3, TAS2R30, DNAH3, KIAA0040, NDUFS7, SSPO* and* UBXN11*.^65^ This study also suggested that NF1 cNFs, which can occur in the 10s–1000s in an individual patient, exhibit different critical molecular changes than sporadic *NF1*^*+/+*^ cNFs, which generally occur in isolation.^65^ Specifically, KIR2DL5 (a KIR-family receptor with no known ligand) mutation and/or loss of expression, which is only observed only in sporadic cNFs, serves to promote cellular proliferation in human Schwann cells.^65^ Other studies of cNF have observed signs of microsatellite instability with no accompanying somatic mismatch repair gene mutations in cNF tumour samples, and have observed only a few cNF tumours with somatic mutations in *TP53* and *RB1*.^53,54^

Studies of gene copy number variation (CNV) in cNFs using array comparative genome hybridisation (aCGH) have observed that there is a scarcity of CNVs in cNF or other dermal (subcutaneous) neurofibromas.^66–69^ Beyond *NF1* deletion, only one of these studies observed any CNVs in cNF, and this was a gain of *CCND1*.^66^ A more recent study identified a small number of regions that had chromosomal imbalances in nine cNFs from independent NF1 patients; 13 regions were decreased (33–76% of patients, depending on the region), and 3 were increased (44–76% of patients).^70^ Of the genes in these regions, the expression of *UST* and *ARC* were identified as significantly correlated with CNVs.^70^ In summary, very few non-*NF1* genomic variants associated with cNF formation and growth are currently known, and future studies are needed to identify other non-*NF1* variants that drive cNF formation. Furthermore, for the non-*NF1* variants that have so far been observed in cNF, mechanistic studies may be valuable in determining their roles as potential drivers or functional modifiers in cNF development. For example, several mutations observed by Anastasaki et al. in NF1 cNFs such as *MAML3* c.1513_1514del have also been observed in bona fide cancers, suggesting that they may be biologically meaningful mutations.^65^

A further gap in our knowledge of cNF development is a paucity of RNA expression data sets with matched normal nerve sheath tissue; however, generation of such a data set may suffer from logistic and ethical challenges. In this respect, animal models of cNF may provide an alternative source of tissue for genomic characterisation. Another approach may emulate the methodology currently employed by Steensma and colleagues. In this ongoing trial, the authors are obtaining cutaneous neurofibroma samples and patient-matched skin samples.^71^ Similarly controlled future translational studies may provide additional insight into novel genomic aspects of cNF biology. In designing such projects, it is important to consider the use of current genomics methodology such as single-cell RNA and whole-genome sequencing. These approaches may facilitate the exploration of the cellular and genomic heterogeneity of cNF, and this could provide a greater understanding of the relative contributions and roles of various cell types in cNF formation.

Another aspect of genomic perturbation in cNF that has not been well explored are mechanisms of genomic regulation such as DNA methylation, histone methylation and microRNA expression. Although there are currently no studies comparing gene/miRNA expression or methylation in cNF with unaffected normal tissue such as myelinated nerve tissue, several studies have compared methylation and RNA/miRNA expression patterns in cNF to other NF-related tumour types.^72,73^ These studies identified significant differences between cNFs, pNFs and MPNSTs, with respect to their DNA methylation, miRNA and gene expression profiles. Other genomics-driven studies have demonstrated a clear role for methylation and transcriptional dysregulation in NF1-linked tumours.^74,75^ Continued research in this area is critical to gain a comprehensive picture of the genomic landscape of cNF to guide future therapeutic development.

### The microenvironment of cNF

Cutaneous neurofibromas have a complex and poorly understood microenvironment comprising multiple cell types, including NF1-deficient proliferative Schwann cells, macrophages, mast cells, fibroblasts and neurons, as well as blood vessels and a collagenous matrix (Fig. 2b). While many cellular components of cNF are not well studied, a role has been demonstrated for mast cells in neurofibroma growth/maintenance. These cells, which are a component of the innate immune system, are observed in several types of NF1-related tumours, including cNFs.^25,76^ Mast cells are thought to serve a complex pro-tumour role in these diseases.^77^ The presence of mast cells in cNFs was first described by Greggio in 1911, and it was later hypothesised that they may be an important element of the cNF microenvironment.^25,78^ Other studies have clarified the role of mast cells in cNF growth. Yang et al. demonstrated that homozygous deletion of *Nf1* in Schwann cells caused excessive secretion of Kit ligand (SCF), a signalling molecule that activates mast cells. They also discovered that, in comparison to *Nf1* wild-type mast cells, *Nf1*^+/−^ mast cells are hyper-responsive to Kit ligand.^79^ This hyperactivation is associated with Ras-mediated PI3K signalling, indicating that *Nf1*^+/−^ mast cells are uniquely affected by *Nf1*^−/−^ Schwann cell signalling mechanisms.^79^ Mast cells may stimulate microenvironmental cNF changes by inducing the formation of a collagenous matrix by transforming growth factor beta (TGF-β)-mediated stimulation of cNF fibroblasts, and by secretion of other molecules such as platelet-derived growth factor (PDGF), fibroblast growth factor (FGF), vascular endothelial growth factor (VEGF) and histamine.^25,80^ In mouse models of cNF, studies demonstrated that cNFs contain infiltrating mast cells.^24,30^ A study of human cNF found that these tumours contain a greater abundance of mast cells and lymphatic vessels, as well as a larger lymphatic vessel diameter than several other NF-associated tumour types, thus suggesting that mast cells play a role in human cNF formation or growth.^81^

The relationship between other cell types and cNF growth is poorly understood. Macrophages are reported to be present in cNF and are abundant in pNFs.^23,26,82,83^ While the associations between macrophages and both NF1-associated mouse and human pNFs has been investigated, similar studies have not yet been performed in cNF.^26,83^ A genomics-focused approach may help to address the potential associations between the microenvironment, mutation burden and tumour growth. Genomic approaches to the deconvolution of microenvironmental cellular subtypes are increasingly utilised in oncology research; for example, methods such as CIBERSORT and TIMER have been used to characterise the diversity of tumour immune cell infiltrate populations across many cancer types.^84,85^ Applying these approaches to cNF data sets may uncover new details about these tumours and the role of the microenvironment.

### Endocrine signalling and cNF

Endocrine signalling is thought to be an important factor in cNF growth and development.^30,86,87^ This hypothesis is underpinned by the observation of the rapid onset and growth of neurofibromas that can occur during puberty and pregnancy,^17,86,88,89^ and there are increasing amounts of molecular data to support this idea. The puberty-related increases in cNF growth may be due to changes in endocrine signalling;^90^ cNFs from NF1 patients have increased immunohistochemical staining for growth hormone receptor (GHR), compared with sporadic cNF and the ligand for GHR is increased during normal puberty.^90^ Growth hormone (GH) hypersecretion has been observed in some NF1 patients, but other studies have concluded that NF1 patients have GH deficiency,^91–93^ and thus the interplay between GH and cNF growth remains unclear at present.

It is well documented that NF1-associated cNFs are frequently progesterone receptor positive, although they are infrequently oestrogen receptor positive.^87,94^ McLaughlin and Jacks observed that the progesterone receptor is not expressed by *NF1*^*−/−*^ Schwann cells in cNF, but rather by other cNF-associated cells that are S100-negative.^94^ One possibility proposed by the authors is that these cells are fibroblasts, perineurial cells, *NF1*^*+/−*^ Schwann cells or *NF1* wild-type Schwann cells.^94^ Others have found that neurofibroma-derived Schwann cells can proliferate in response to progesterone or oestrogen treatment in vitro.^95,96^ In vivo mouse xenograft experiments with cNF-derived Schwann cells demonstrated that one of four tumours treated with oestrogen grew, while another one of four tumours treated with progesterone grew, suggesting that the presence of oestrogen or progesterone can affect cNF growth.^97^ However, a study of 59 NF1 patients taking hormonal contraceptives observed that patients taking either oestrogen–progestogen or progestogen had no associated tumour growth;^2^ although, tumour growth was reported by two patients taking high-dose synthetic progesterone. Collectively, these data suggest that further research is needed to determine the role of progesterone on cNF tumour growth, and to elucidate the cNF cell population(s) most affected by progesterone receptor activation.^2^ Such future research could be enhanced with genomics approaches; for example, using a method such as single-sample gene-set enrichment analysis to detect oestrogen signalling pathways in cNFs, similar to a study that performed the same in colorectal cancer samples.^98^

### Current and emerging treatment paradigms for cNF

There is currently no effective therapy for cNFs, and the mainstay of clinical management involves monitoring and informing the patients about their prognosis and the nature of their tumours. Treatment options for cNFs are limited to elective surgical approaches or laser-based ablation of cNFs.^99,100^ Other techniques have been applied for the removal of cNFs, including electrodessication and radiofrequency ablation.^100–102^ Surgical/ablative approaches involve a risk of scarring and skin discoloration,^100,103^ and surgical approaches are not practical in patients with numerous cNFs. These clinical deficiencies emphasise a need for novel, genomically guided therapies that are based on a greater understanding of the molecular and cellular mechanisms that underlie NF1-associated cNFs, and at the time of writing, nine interventional clinical trials were identified for cNFs (Table 1). Of these, five have been completed, three are active and not recruiting and one is currently active and recruiting.

**Table 1:** Clinical trials for cutaneous neurofibromas (arranged chronologically by first received date)

ClinicalTrials.gov ID	Study name	Recruitment status	Intervention	Mechanism	Phase	First received	Citation
NCT03090971	Use of topical liquid diclofenac following laser microporation of cutaneous neurofibromas in patients with NF1	Active, not recruiting	Diclofenac sodium	NSAID	Phase 2	March 2017	^112^
NCT02839720	Selumetinib in treating patients with neurofibromatosis type 1 and dermal neurofibroma	Not yet recruiting	Selumetinib	MEK	Phase 2	July 2016	^128^
NCT02728388	Photodynamic therapy for benign dermal neurofibromas	Recruiting	Levulan kerastick	Photosensitizer	Phase 2	March 2016	^110^
NCT02332902	Everolimus for treatment of disfiguring cutaneous lesions in NF1	Completed	Everolimus	mTOR	Phase 2	December 2014	^124^
NCT01682811	Photodynamic therapy (PDT) for benign dermal neurofibromas (NF1)	Active, not recruiting	Levulan (5-aminolevulinic acid)	Photosensitizer	Phase 1	September 2012	^109^
NCT01412892	Use of RAD001 as monotherapy in the treatment of neurofibromatosis 1 related internal plexiform neurofibromas	Completed	RAD001 (everolimus)	mTOR	Phase 2	August 2011	^132^
NCT01031901	Topical rapamycin therapy to alleviate cutaneous manifestations of tuberous sclerosis complex (TSC) and neurofibromatosis I (NF1)	Completed	Skinercity plus sirolimus (rapamycin)	mTOR	Phase 1	December 2009	^133^
NCT00865644	Topical imiquimod 5% cream for treatment of cutaneous neurofibromas in adults with NF1	Completed	Imiquimod 5% cream	TLR 7/8	Phase 1	March 2009	^105^
NCT00657202	Ranibizumab for neurofibromas associated with NF1	Completed	Ranibizumab (lucentis)	VEGF	Early Phase 1	March 2008	^121^

Many modern clinical cNF trials rely on specific molecular targets that are relevant to NF1 tumour biology. Oncogenomics-oriented studies that identify key molecular mediators of cNF growth and development will aid the selection of interventions for future cNF clinical trials

Three cNF-specific clinical trials have evaluated the effect of topically applied treatments on cNF. Topical administration offers the benefit of limiting the systemic exposure; however, it can make effective drug delivery more difficult, as the skin acts as a barrier to most drugs.^104^ A second potential challenge for topical administration of cNF treatments concerns the potential impracticality for patients with extensive lesions, who may find it unfeasible to regularly apply a treatment to 40–70% of their body surface. Furthermore, no successful results have yet been reported for topically applied monotherapies in cNF (for example, imiquimod).^105^ Other reports have indicated that off-label topical application of ketotifen may be used to prevent cNF growth or reduce cNF burden by blocking the degranulation of mast cells in cNFs^106–108^; however, this drug has not seen widespread clinical adoption or success for treating cNF.

Beyond topical monotherapies, other trials have been initiated to study the safety and efficacy of photodynamic therapy (PDT) on cNFs. PDT involves pretreatment of a skin lesion with a photosensitising agent such aminolevulinic acid (ALA) followed by light exposure, to induce localised cell death. A current Phase 1 study is exploring the use of topical ALA and red light as a cNF intervention,^109^ and preliminary results have indicated that PDT may slow the growth of cNFs in adults. Applying PDT to developing cNFs during adolescence may be an effective preventative therapeutic measure, and a Phase 2 study will investigate whether this intervention will slow the growth of cNFs in adolescents.^110^ Other trials are investigating the direct injection of active agents into cNFs. One such study assessed diclofenac, a nonsteroidal anti-inflammatory drug, in cNF patients.^111^ The study did not show a conclusive change in neurofibroma size; in some cases, the neurofibromas grew, while in other cases the cNFs presented with necrosis and detached. These results appear to serve as a proof-of-concept for a study that will investigate topical diclofenac following laser microporation of cNFs.^112^

Since cNFs are reported to be composed of the same cell types found in the peripheral nerves, their biology may be similar to tumours like pNFs.^113,114^ Most of the interventional clinical trials described for cNFs stem from therapies under development for other manifestations of NF1 (Table 1). The use of targeted therapeutics such as VEGF, mTOR, c-kit or MEK inhibitors in cNF is likely based on data suggesting that these targets are important in NF1-deficient tumours.^115–118^ Additionally, the overlap between pathways dysregulated by NF1 loss and pathways dysregulated in other cancer types has also inspired clinical trials that reposition existing cancer treatment options. An example of such a target is vascular endothelial growth factor (VEGF), an angiogenic signalling molecule that is highly expressed in cNFs and other NF1-linked tumours.^119,120^ A trial investigated the potential for VEGF as a viable target by directly injecting the VEGF inhibitor ranibizumab into cNFs.^121^ The primary outcomes of the trial were changes to cNF volume and interstitial pressure. Patients served as their own controls, with three tumours treated with a single injection of ranibizumab and three tumours treated with saline controls. Results are not yet reported for this study. The mechanistic target of rapamycin (mTOR) is another target with relevance to NF1 tumour biology.^115^ This protein has previously been studied in pNF and MPNST-focussed clinical trials, using the mTOR inhibitor sirolimus as well as the mTOR inhibitor everolimus in combination with the VEGF inhibitor bevacizumab.^122,123^ However, the results from these studies indicated that these interventions were inactive in pNF and MPNST, respectively. In cNF, a single-arm interventional trial assessed the effect of everolimus on cNF growth.^124^ As with the studies of mTOR inhibitors in pNF and MPNST, this trial observed no change in cNF growth over the course of the intervention. It is possible that this is due to the stability of untreated cNFs over time, or due to the intervention preventing further growth of the cNFs. Additionally, there was no control arm for this study, so it remains to be confirmed whether mTOR inhibition is effective in reducing cNF burden.

Mast/stem cell growth factor receptor kit (c-kit), a cytokine receptor found on mast cells that is activated by SCF, was identified as a critical molecular component in NF1-deficient mast cells and tumours.^25,76,125^ One strategy for targeting tumours with mast cell components such as cNFs and pNFs is imatinib-mediated inhibition of c-kit. In NF1 patients with pNF, a Phase 2 clinical trial demonstrated response in 6/36 (17%) of participants.^76^ Changes in cNF size were not tracked in these patients, but this study may provide a model for designing a cNF-focussed clinical trial with imatinib. In a separate case report studying the effect of imatinib in an NF1 patient with cutaneous vasculopathy, the authors reported no change in the volume of the patient’s cNFs;^126^ however, the intervention was prematurely discontinued due to the development of adverse side effects.^126^ A clinical trial with multiple patients would be required to make any further conclusions about the effect of c-kit inhibition on cNFs.

Mitogen-activated protein kinase kinase (MAPK2/MEK), an integral signalling component of the Ras pathway, is a compelling target in many NF1-related tumours. The MEK inhibitor selumetinib has been reported as a low-toxicity and efficacious intervention in a trial for children with NF1 and inoperable pNFs.^116,127^ While trials of MEK inhibitors in cNF are yet to be conducted, the effect of selumetinib on cNF size as well as p-ERK and p-AKT levels will be explored in a recently initiated clinical trial.^128^ It remains to be seen whether selumetinib treatment is effective in cNF.

While MEK inhibition may be demonstrated to be a successful therapeutic approach for cNF, the development of new cNF trials is partially dependent on the identification of novel and pharmacologically tractable molecular targets with critical relevance to NF1 tumour biology. We propose that the application of modern oncogenomic approaches to cNF may yield valuable insights and allow the identification of putative drug targets to improve the therapeutic prospects of NF1 patients.

## knowledge gaps and prospective avenues for future study

Cutaneous neurofibromas are a significant burden for individuals with NF1.^15,16,19^ As such, improved prevention and treatment of cNF is an important aspect in raising the quality of life for these patients. The observation that cNFs can undergo rapid proliferation followed by quiescence presents a challenge and an opportunity.^21^ The possibility that quiescent cNFs may not respond to anti-proliferative drugs is problematic; on the other hand, there is an opportunity to understand the mechanism underlying the quiescent process, and thus to identify new therapeutic targets. This may also inform the field of biological differences to consider when developing prophylactic therapeutic strategies, as opposed to cNF treatments. While the field has elucidated several aspects of cNF aetiology and pathology, most of the mechanisms involved in the evolution of these tumours remain unknown. Somatic and germline *NF1* mutations are diverse, and for most cases the genotype–phenotype relationship, with respect to *NF1* mutation and cNF burden, is not straightforward.

Further investigations of cNF biology are needed to facilitate the identification of novel therapeutic targets. It is not known which intracellular signalling mechanisms are most responsible for cNF formation, the specific mechanisms by which mast cells promote cNF formation, and the contribution, or lack thereof, of macrophages and other components of the tumour microenvironment to cNF growth and development. The question of whether pharmacologic targeting of these components in cNF is a viable therapeutic approach is currently being investigated, and may yield more effective or more convenient therapeutic interventions for patients with cNF.

It is also unclear which genetic factors, beyond *NF1*, are responsible for cNF growth and progression. Epigenetic regulatory mechanisms are also thought to be involved in the development of other NF1-linked tumours; these or other epigenetic mechanisms may also be dysregulated in cNF.^74,75^ While many large-scale resources exist for exploring the mutational landscape of malignant tumours (e.g., TCGA, cBioPortal, Project GENIE), there are limited genomic explorations of neurofibromas. To address this, the Children’s Tumour Foundation created a cutaneous neurofibroma data resource.^63^ This resource contains whole-genome sequencing and SNP microarray data from cNFs and patient-matched blood samples, as well as cNF RNA sequencing data.^63^ The data are open access and can be found on Sage Bionetworks’ Synapse platform (https://www.synapse.org/cutaneousNF). Other similar publicly available resources include microarray-based RNA expression data from cNF-derived Schwann cell cultures and cNF patient samples (Gene Expression Omnibus, GSE32029, GSE14038, GSE66743), and cNF aCGH data (GSE58000).

Progress is being made by the neurofibromatosis research community towards addressing the current knowledge gaps with different resources available for cNF research (Table 2). As part of these efforts, cNF research should leverage the power of current technologies developed for oncology research. Newer, faster and less costly techniques for genomic and transcriptomic profiling as well as sharing of the resulting data is an important approach to facilitating this type of research. Integrated genomics approaches, such as the simultaneous analysis of mutational and RNA expression data, can be used to identify novel cancer driver genes, or uncover critical cancer signalling mechanisms.^129–131^ In addition, the previously described immune characterisation techniques can be used for pan-cancer analysis to discover previously unknown features of the tumour microenvironment. By applying these methods in cNF research, we may be able to accelerate the pace with which novel therapies for cNFs are identified and address a major life-long burden for NF1 patients.

**Table 2:** Resources for cutaneous neurofibroma research

Resource	Resource type	URL
Neurofibromatosis Therapeutic Acceleration Program (NTAP) cNF Initiative	Funding agency, working group	http://www.n-tap.org/nf1-and-cutaneous-neurofibroma/
Children’s Tumor Foundation (CTF)	Funding agency, tissue repository	http://www.ctf.org/
Giorgio Foundation	Funding agency, research consortium	https://endnf1.org/
Congressionally Directed Medical Research Programmes Neurofibromatosis Research Program (CDMRP NFRP)	Funding agency, reagent repository	http://cdmrp.army.mil/nfrp/
CTF cNF Data Resource	Data repository	https://www.synapse.org/cutaneousNF
Response Evaluation in Neurofibromatosis and Schwannomatosis (REiNS) cNF Collaboration	Working group	https://ccrod.cancer.gov/confluence/display/REINS/Cutaneous+Neurofibromas

Funding agencies, working groups, consortia and publicly accessible repositories of data and reagents are included

## References

[1] Evans, D. G. et al. Birth incidence and prevalence of tumor-prone syndromes: estimates from a UK family genetic register service. *Am. J. Med. Genet. A* **152A**, 327–332 (2010). 10.1002/ajmg.a.33139 20082463

[2] Lammert, M., Friedman, J. M., Kluwe, L. & Mautner, V. F. Prevalence of neurofibromatosis 1 in German children at elementary school enrollment. *Arch. Dermatol*. **141**, 71–74 (2005). 10.1001/archderm.141.1.71 15655144

[3] Boyd KP, Korf BR, Theos A (2009). Neurofibromatosis type 1. J. Am. Acad. Dermatol..

[4] Friedman, J. Neurofibromatosis 1 [Internet]. Gene reviews. University of Washington. http://www.ncbi.nlm.nih.gov/pubmed/20301288. Accessed 6 July 2016 (2014).

[5] Recklinghausen, F. von. Ueber die multiplen Fibrome der Haut und ihre Beziehung zu den multiplen Neuromen [Internet]. https://wellcomelibrary.org. Accessed 8 August 2017 (1882).

[6] Wallace MR (1991). A de novo Alu insertion results in neurofibromatosis type 1. Nature.

[7] Viskochil D (1990). Deletions and a translocation interrupt a cloned gene at the neurofibromatosis type 1 locus. Cell.

[8] Cawthon RM (1990). A major segment of the neurofibromatosis type 1 gene: cDNA sequence, genomic structure, and point mutations. Cell.

[9] Plotkin SR (2012). Quantitative assessment of whole-body tumor burden in adult patients with neurofibromatosis. PLoS ONE.

[10] Tonsgard JH, Kwak SM, Short MP, Dachman AH (1998). CT imaging in adults with neurofibromatosis-1: frequent asymptomatic plexiform lesions. Neurology.

[11] Evans DGR (2002). Malignant peripheral nerve sheath tumours in neurofibromatosis 1. J. Med. Genet..

[12] Ingham S (2011). Malignant peripheral nerve sheath tumours in NF1: improved survival in women and in recent years. Eur. J. Cancer.

[13] Sabbagh A (2009). Unravelling the genetic basis of variable clinical expression in neurofibromatosis 1. Hum. Mol. Genet..

[14] Jouhilahti EM (2011). The development of cutaneous neurofibromas. Am. J. Pathol..

[15] Peltonen, J., Jouhilahti, E.-M. & Peltonen, S. in *Neurofibromatosis Type 1* (eds Upadhyaya, M. & Cooper, D.) 393–403 (Springer, Berlin, Heidelberg, 2012).

[16] Ferner RE (2006). Guidelines for the diagnosis and management of individuals with neurofibromatosis 1. J. Med. Genet..

[17] Dugoff L, Sujansky E (1996). Neurofibromatosis type 1 and pregnancy. Am. J. Med. Genet..

[18] Wu BL, Austin MA, Schneider GH, Boles RG, Korf BR (1995). Deletion of the entire NF1 gene detected by FISH: four deletion patients associated with severe manifestations. Am. J. Med. Genet..

[19] Granström S, Langenbruch A, Augustin M, Mautner VF (2012). Psychological burden in adult neurofibromatosis type 1 patients: impact of disease visibility on body image. Dermatology.

[20] Rodriguez FJ, Folpe AL, Giannini C, Perry A (2012). Pathology of peripheral nerve sheath tumors: diagnostic overview and update on selected diagnostic problems. Acta Neuropathol..

[21] Ruggieri, M., Upadhyaya, M., Rocco, C. Di, Gabriele, A. & Pascual-Castroviejo, I. in *Neurocutaneous Disorders Phakomatoses and Hamartoneoplastic Syndromes* (eds Ruggieri, M., Castroviejo, I. P. & Rocco, C. D.) 51–151 (Springer, Vienna, 2008).

[22] Ortiz-Hidalgo, C. & Weller, R. in *Histology for Pathologists* 2nd edn (ed. Sternberg, S.) 285–311 (Lippincott-Raven Publishers, Philadelphia, 1997).

[23] Le LQ, Kesterson RA, Gutmann DH (2016). Defining the research landscape for dermal neurofibromas. Oncol. Times.

[24] Liao CP (2016). The role of nerve microenvironment for neurofibroma development. Oncotarget.

[25] Yang FC (2006). Nf1+/- mast cells induce neurofibroma like phenotypes through secreted TGF-beta signaling. Hum. Mol. Genet..

[26] Choi K (2017). An inflammatory gene signature distinguishes neurofibroma Schwann cells and macrophages from cells in the normal peripheral nervous system. Sci. Rep..

[27] Hirose T (2003). Immunohistochemical demonstration of EMA/Glut1-positive perineurial cells and CD34-positive fibroblastic cells in peripheral nerve sheath tumors. Mod. Pathol..

[28] Riddle ND, Gorden L, Rojiani MV, Hakam A, Rojiani AM (2010). CD44 and p53 immunoexpression patterns in NF1 neoplasms - indicators of malignancy and infiltration. Int. J. Clin. Exp. Pathol..

[29] Wu J (2008). Plexiform and dermal neurofibromas and pigmentation are caused by Nf1 loss in desert hedgehog-expressing cells. Cancer Cell.

[30] Le, L. Q., Shipman, T., Burns, D. K. & Parada, L. F. Cell of origin and microenvironment contribution for NF1-associated dermal neurofibromas. *Cell Stem Cell* **4**, 453–463 (2009). 10.1016/j.stem.2009.03.017 PMC2737469 19427294

[31] (NHGRI) National Human Genome Research Institute. [NCT00314119] *Natural History and Biology of Skin Neurofibromas in Neurofibromatosis Type 1.* ClinicalTrials.gov. (2006).

[32] National Cancer Institute (NCI). [NCT00924196] *Natural History Study of Patients with Neurofibromatosis Type I*. ClinicalTrials.gov. (2009).

[33] Ratner N, Miller SJ (2015). A RASopathy gene commonly mutated in cancer: the neurofibromatosis type 1 tumour suppressor. Nat. Rev. Cancer.

[34] Eccleston JF, Moore KJ, Morgan L, Skinner RH, Lowe PN (1993). Kinetics of interaction between normal and proline 12 Ras and the GTPase-activating proteins, p120-GAP and neurofibromin. The significance of the intrinsic GTPase rate in determining the transforming ability of ras. J. Biol. Chem..

[35] Downward J (2003). Targeting RAS signalling pathways in cancer therapy. Nat. Rev. Cancer.

[36] Philpott C, Tovell H, Frayling IM, Cooper DN, Upadhyaya M (2017). The NF1 somatic mutational landscape in sporadic human cancers. Hum. Genomics.

[37] Scholz, S. et al. NF1 mutations in conjunctival melanoma. *Br. J. Cancer*. (2018). 10.1038/s41416-018-0046-5. 10.1038/s41416-018-0046-5 PMC5943412 29559732

[38] Ars E (2003). Recurrent mutations in the NF1 gene are common among neurofibromatosis type 1 patients. J. Med. Genet..

[39] van Minkelen R (2014). A clinical and genetic overview of 18 years neurofibromatosis type 1 molecular diagnostics in the Netherlands. Clin. Genet..

[40] Upadhyaya M (2006). The heterogeneous nature of germline mutations in NF1 patients with malignant peripheral serve sheath tumours (MPNSTs). Hum. Mutat..

[41] Upadhyaya M (2008). Germline and somatic N*F1* gene mutations in plexiform neurofibromas. Hum. Mutat..

[42] Raponi M, Upadhyaya M, Baralle D (2006). Functional splicing assay shows a pathogenic intronic mutation in neurofibromatosis type 1 (NF1) due to intronic sequence exonization. Hum. Mutat..

[43] Pasmant E (2010). NF1 microdeletions in neurofibromatosis type 1: from genotype to phenotype. Hum. Mutat..

[44] De Raedt, T. et al. Somatic loss of wild type NF1 allele in neurofibromas: comparison of NF1 microdeletion and non-microdeletion patients. *Genes Chromosomes Cancer* **45**, 893–904 (2006). 10.1002/gcc.20353 16830335

[45] Kehrer-Sawatzki H, Schmid E, Fünsterer C, Kluwe L, Mautner VF (2008). Absence of cutaneous neurofibromas in an NF1 patient with an atypical deletion partially overlapping the common 1.4 Mb microdeleted region. Am. J. Med. Genet. A.

[46] Kehrer-Sawatzki H, Mautner VF, Cooper DN (2017). Emerging genotype-phenotype relationships in patients with large NF1 deletions. Hum. Genet..

[47] Mautner VF (2010). Clinical characterisation of 29 neurofibromatosis type-1 patients with molecularly ascertained 1.4 Mb type-1 NF1 deletions. J. Med. Genet..

[48] Hinman MN, Sharma A, Luo G, Lou H (2014). Neurofibromatosis type 1 alternative splicing is a key regulator of Ras signaling in neurons. Mol. Cell Biol..

[49] Lenarduzzi M (2013). MicroRNA-193b enhances tumor progression via down regulation of neurofibromin 1. PLoS ONE.

[50] Cichowski K, Santiago S, Jardim M, Johnson BW, Jacks T (2003). Dynamic regulation of the Ras pathway via proteolysis of the NF1 tumor suppressor. Genes Dev..

[51] Yap YS (2014). The NF1 gene revisited - from bench to bedside. Oncotarget.

[52] Emmerich D (2015). Somatic neurofibromatosis type 1 (NF1) inactivation events in cutaneous neurofibromas of a single NF1 patient. Eur. J. Hum. Genet..

[53] Thomas L, Kluwe L, Chuzhanova N, Mautner V, Upadhyaya M (2010). Analysis of NF1 somatic mutations in cutaneous neurofibromas from patients with high tumor burden. Neurogenetics.

[54] Thomas L (2012). Exploring the somatic NF1 mutational spectrum associated with NF1 cutaneous neurofibromas. Eur. J. Hum. Genet..

[55] Upadhyaya M (2007). An absence of cutaneous neurofibromas associated with a 3-bp inframe deletion in exon 17 of the NF1 gene (c.2970-2972 delAAT): evidence of a clinically significant NF1 genotype-phenotype correlation. Am. J. Hum. Genet..

[56] Pinna V (2014). p.Arg1809Cys substitution in neurofibromin is associated with a distinctive NF1 phenotype without neurofibromas. Eur. J. Hum. Genet..

[57] Santoro C (2015). Arg(1809) substitution in neurofibromin: further evidence of a genotype-phenotype correlation in neurofibromatosis type 1. Eur. J. Hum. Genet..

[58] Saito H, Yoshida T, Yamazaki H, Suzuki N (2007). Conditional N-rasG12V expression promotes manifestations of neurofibromatosis in a mouse model. Oncogene.

[59] Sherman LS, Atit R, Rosenbaum T, Cox AD, Ratner N (2000). Single cell Ras-GTP analysis reveals altered Ras activity in a subpopulation of neurofibroma Schwann cells but not fibroblasts. J. Biol. Chem..

[60] Messiaen L (2009). Clinical and mutational spectrum of neurofibromatosis type 1-like syndrome. JAMA.

[61] Way GP (2016). A machine learning classifier trained on cancer transcriptomes detects NF1 inactivation signal in glioblastoma. BMC Genomics.

[62] Bhoj, E. J. et al. Phenotypic predictors and final diagnoses in patients referred for RASopathy testing by targeted next-generation sequencing. *Genet. Med.* **19**, 715–718 (2016). 10.1038/gim.2016.169 PMC6095193 27763634

[63] Gosline SJC (2017). A high-throughput molecular data resource for cutaneous neurofibromas. Sci. Data.

[64] Faden DL, Asthana S, Tihan T, De Risi J, Kliot M (2017). Whole exome sequencing of growing and non-growing cutaneous neurofibromas from a single patient with neurofibromatosis type 1. Gao J-X, editor. PLoS ONE.

[65] Anastasaki, C. et al. KIR2DL5 mutation and loss underlies sporadic dermal neurofibroma pathogenesis and growth. *Oncotarget* **8**, 47574–47585 (2017). 10.18632/oncotarget.17736 PMC5564588 28548933

[66] Mantripragada KK (2008). High-resolution DNA copy number profiling of malignant peripheral nerve sheath tumors using targeted microarray-based comparative genomic hybridization. Clin. Cancer Res..

[67] Mantripragada, K. K et al. Genome-wide high-resolution analysis of DNA copy number alterations in NF1-associated malignant peripheral nerve sheath tumors using 32K BAC array. *Genes Chromosomes Cancer* **48**, 897–907 (2009). 10.1002/gcc.20695 19603524

[68] Brekke HR (2010). Genomic changes in chromosomes 10, 16, and X in malignant peripheral nerve sheath tumors identify a high-risk patient group. J. Clin. Oncol..

[69] Beert, E. et al. Atypical neurofibromas in neurofibromatosis type 1 are premalignant tumors. *Genes Chromosomes Cancer* **50**,1021–1032 (2011). 10.1002/gcc.20921 21987445

[70] Asai A (2015). High-resolution 400K oligonucleotide array comparative genomic hybridization analysis of neurofibromatosis type 1-associated cutaneous neurofibromas. Gene.

[71] Steensma, M. [NCT02777775] *Targeting the Mechanisms Underlying Cutaneous Neurofibroma Formation in NF1: A Clinical Translational Approach*. ClinicalTrials.gov. (2016).

[72] Masliah-Planchon J (2013). MicroRNAome profiling in benign and malignant neurofibromatosis type 1-associated nerve sheath tumors: evidences of PTEN pathway alterations in early NF1 tumorigenesis. BMC Genomics.

[73] Röhrich M (2016). Methylation-based classification of benign and malignant peripheral nerve sheath tumors. Acta Neuropathol..

[74] De Raedt T (2014). PRC2 loss amplifies Ras-driven transcription and confers sensitivity to BRD4-based therapies. Nature.

[75] Lee W (2014). PRC2 is recurrently inactivated through EED or SUZ12 loss in malignant peripheral nerve sheath tumors. Nat. Genet..

[76] Robertson KA (2012). Imatinib mesylate for plexiform neurofibromas in patients with neurofibromatosis type 1: a phase 2 trial. Lancet Oncol..

[77] Maciel TT, Moura IC, Hermine O (2015). The role of mast cells in cancers. F1000Prime Rep..

[78] Greggio H (1911). Les cellules granuleuses (Mastzellen) dans les tissus normaux et dans certaines maladies chirurgicales. Arch. Méd. Exp..

[79] Feng-Chun Y (2003). Neurofibromin-deficient Schwann cells secrete a potent migratory stimulus for Nf1+/– mast cells. J. Clin. Invest..

[80] Viskochil DH (2003). It takes two to tango: mast cell and Schwann cell interactions in neurofibromas. J. Clin. Invest..

[81] Friedrich RE, Naber U, Glatzel M, Hagel C (2015). Vessel and mast cell densities in sporadic and syndrome-associated peripheral nerve sheath tumors. Anticancer Res..

[82] Takata M, Imai T, Hirone T (1994). Factor-XIIIa-positive cells in normal peripheral nerves and cutaneous neurofibromas of type-1 neurofibromatosis. Am. J. Dermatopathol..

[83] Prada CE (2013). Neurofibroma-associated macrophages play roles in tumor growth and response to pharmacological inhibition. Acta Neuropathol..

[84] Newman AM (2015). Robust enumeration of cell subsets from tissue expression profiles. Nat. Methods.

[85] Li, B. et al. Comprehensive analyses of tumor immunity: implications for cancer immunotherapy. *Genome Biol*. **17**, 174 (2016). 10.1186/s13059-016-1028-7 PMC4993001 27549193

[86] Sharpe JC, Young RH (1936). Neurofibromatosis: the effect of pregnancy on the skin manifestations. J. Am. Med. Assoc..

[87] Geller M (2008). Progesterone and estrogen receptors in neurofibromas of patients with NF1. Clin. Med. Pathol..

[88] Cesaretti C (2013). Neurofibromatosis type 1 and pregnancy: maternal complications and attitudes about prenatal diagnosis. Am. J. Med. Genet. A..

[89] Rasmussen SA, Friedman JM (2000). NF1 gene and neurofibromatosis 1. Am. J. Epidemiol..

[90] Cunha KSG, Barboza EP, Fonseca EC (2003). Identification of growth hormone receptor in localized neurofibromas of patients with neurofibromatosis type 1. J. Clin. Pathol..

[91] Bizzarri C, Bottaro G (2015). Endocrine implications of neurofibromatosis 1 in childhood. Horm. Res. Paediatr..

[92] Howell SJ, Wilton P, Lindberg A, Shalet SM (2000). Growth hormone and neurofibromatosis. Horm. Res..

[93] Vassilopoulou-Sellin R, Klein MJ, Slopis JK (2000). Growth hormone deficiency in children with neurofibromatosis type 1 without suprasellar lesions. Pediatr. Neurol..

[94] McLaughlin ME, Jacks T (2003). Progesterone receptor expression in neurofibromas. Cancer Res..

[95] Overdiek A, Winner U, Mayatepek E, Rosenbaum T (2008). Schwann cells from human neurofibromas show increased proliferation rates under the influence of progesterone. Pediatr. Res..

[96] Fishbein L (2007). In vitro studies of steroid hormones in neurofibromatosis 1 tumors and schwann cells. Mol. Carcinog..

[97] Li H (2010). Analysis of steroid hormone effects on xenografted human NF1 tumor schwann cells. Cancer Biol. Ther..

[98] Liu, D. Gene signa tures of estrogen and progesterone receptor pathways predict the prognosis of colorectal cancer. *FEBS J*. **283**, 3115–3133 (2016). 10.1111/febs.13798 27376509

[99] Chiang YZ, Al-Niaimi F, Ferguson J, August PJ, Madan V (2012). Carbon dioxide laser treatment of cutaneous neurofibromas. Dermatol. Ther..

[100] Kriechbaumer LK, Susani M, Kircher SG, Distelmaier K, Happak W (2014). Comparative study of CO2- and Er:YAG laser ablation of multiple cutaneous neurofibromas in von Recklinghausen’s disease. Lasers Med. Sci..

[101] Kim DH (2016). 27.12 MHz radiofrequency ablation for benign cutaneous lesions. Biomed. Res. Int..

[102] Lutterodt CG, Mohan A, Kirkpatrick N (2016). The use of electrodessication in the treatment of cutaneous neurofibromatosis: A retrospective patient satisfaction outcome assessment. J. Plast. Reconstr. Aesthetic Surg..

[103] Kim SH, Roh SG, Lee NH, Yang KM (2013). Radiofrequency ablation and excision of multiple cutaneous lesions in neurofibromatosis type 1. Arch. Plast. Surg..

[104] Wermeling DP (2008). Microneedles permit transdermal delivery of a skin-impermeant medication to humans. Proc. Natl Acad. Sci. USA.

[105] Massachusetts General Hospital. [NCT00865644] *Topical Imiquimod 5% Cream for Treatment of Cutaneous Neurofibromas in Adults With Neurofibromatosis 1 No Title*. ClinicalTrials.gov. (2009).

[106] Riccardi VM (1987). Mast-cell stabilization to decrease neurofibroma growth. Prelim. Exp. ketotifen. Arch. Dermatol..

[107] Riccardi VM (1993). A controlled multiphase trial of ketotifen to minimize neurofibroma-associated pain and itching. Arch. Dermatol..

[108] Riccardi VM (2015). Ketotifen suppression of NF1 neurofibroma growth over 30 years. Am. J. Med. Genet. A.

[109] Whelan, H. T. [NCT01682811] *Photodynamic Therapy (PDT) for Benign Dermal Neurofibromas (NF1)*. ClinicalTrials.gov. (2012).

[110] Whelan, H. T. [NCT02728388] *Photodynamic Therapy for Benign Dermal Neurofibromas.* ClinicalTrials.gov. (2016).

[111] Geller M (2015). A proof-of-concept assessment of the safety and efficacy of intralesional diclofenac in the treatment of cutaneous neurofibromas. Int. J. Clin. Med..

[112] Fundação Educacional Serra dos Órgãos. [NCT03090971] *Use of Topical Liquid Diclofenac Following Laser Microporation of Cutaneous Neurofibromas in Patients With NF1.* ClinicalTrials.gov. (2017).

[113] Williams VC (2009). Neurofibromatosis type 1 revisited. Pediatrics.

[114] Hirsch NP, Murphy A, Radcliffe JJ (2001). Neurofibromatosis: clinical presentations and anaesthetic implications [Internet]. Br. J. Anaesth..

[115] Johannessen CM (2005). The NF1 tumor suppressor critically regulates TSC2 and mTOR. Proc. Natl Acad. Sci. USA.

[116] Dombi E (2016). Activity of selumetinib in neurofibromatosis type 1–related plexiform neurofibromas. N. Engl. J. Med..

[117] Kawachi Y (2013). N*F1* gene silencing induces upregulation of vascular endothelial growth factor expression in both Schwann and non-Schwann cells. Exp. Dermatol..

[118] Yang FC (2008). Nf1-dependent tumors require a microenvironment containing Nf1+/−- and c-kit-dependent bone marrow. Cell.

[119] Kawachi Y, Xu X, Ichikawa E, Imakado S, Otsuka F (2003). Expression of angiogenic factors in neurofibromas. Exp. Dermatol..

[120] Wasa J (2008). Differential expression of angiogenic factors in peripheral nerve sheath tumors. Clin. Exp. Metastas..

[121] Massachusetts General Hospital. [NCT00657202] *Ranibizumab for Neurofibromas Associated With NF1*. ClinicalTrials.gov. (2008).

[122] Weiss B (2014). Sirolimus for progressive neurofibromatosis type 1-associated plexiform neurofibromas: a neurofibromatosis clinical trials consortium phase II study. Neuro. Oncol..

[123] Widemann BC (2016). SARC016: phase II study of everolimus in combination with bevacizumab in sporadic and neurofibromatosis type 1 (NF1) related refractory malignant peripheral nerve sheath tumors (MPNST). J. Clin. Oncol..

[124] The University of Texas Health Science Center - Houston. [NCT02332902] *Everolimus for Treatment of Disfiguring Cutaneous Lesions in Neurofibromatosis1 CRAD001CUS232T (DCLNF1)*. ClinicalTrials.gov. (2014).

[125] Ingram DA, Yang FC, Travers JB, Clapp DW (1999). Nf1 modulates C-kit signaling in mast cells and neural crest derived melanocytes in vivo in a dose dependent fashion. Pediatr. Res..

[126] Khelifa I, Saurat JH, Prins C (2015). Use of imatinib in a patient with cutaneous vasculopathy in the context of von Recklinghausen disease/neurofibromatosis. Br. J. Dermatol..

[127] National Cancer Institute (NCI). [NCT01362803] *AZD6244 Hydrogen Sulfate for Children With Nervous System Tumors*. ClinicalTrials.gov. (2011).

[128] National Cancer Institute (NCI). [NCT02839720] *Selumetinib in Treating Patients With Neurofibromatosis Type 1 and Dermal Neurofibroma*. ClinicalTrials.gov. (2016).

[129] Peifer M (2012). Integrative genome analyses identify key somatic driver mutations of small-cell lung cancer. Nat. Genet..

[130] Wang K (2014). Whole-genome sequencing and comprehensive molecular profiling identify new driver mutations in gastric cancer. Nat. Genet..

[131] Sivakumar S (2017). Master regulators of oncogenic KRAS. PLoS Med..

[132] Assistance Publique - Hôpitaux de Paris. [NCT01412892] *Use of RAD001 as Monotherapy in the Treatment of Neurofibromatosis 1 Related Internal Plexiform Neurofibromas (NFitor)*. ClinicalTrials.gov. (2011).

[133] The University of Texas Health Science Center - Houston. [NCT01031901] *Topical Rapamycin Therapy to Alleviate Cutaneous Manifestations of Tuberous Sclerosis Complex (TSC) and Neurofibromatosis I (NF1)*. ClinicalTrials.gov. (2009).

